# A longitudinal cohort study of gestational diabetes mellitus and perinatal depression

**DOI:** 10.1186/s12884-022-04667-2

**Published:** 2022-04-19

**Authors:** Haiyan Li, Xiayan Yu, Wenjing Qiang, Mengjuan Lu, Minmin Jiang, Yanyan Hou, Yue Gu, Fangbiao Tao, Beibei Zhu

**Affiliations:** 1 Department of Maternal, Child and Adolescent Health, School of Public Health, Anhui Medical University, No 81 Meishan Road, Hefei, 230032 Anhui China; 2 Key Laboratory of Population Health Across Life Cycle (Anhui Medical University), Ministry of Education of the People’s Republic of China, No 81 Meishan Road, Hefei, 230032 Anhui China; 3 Anhui Provincial Key Laboratory of Population Health and Aristogenics, Anhui Medical University, No 81 Meishan Road, Hefei, 230032 Anhui China; 4 NHC Key Laboratory of Study on Abnormal Gametes and Reproductive Tract, No 81 Meishan Road, Hefei, 230032 Anhui China; 5 Department of Occupational Health and Environmental Health, School of Public Health, Anhui Medical University, No 81 Meishan Road, Hefei, 230032 Anhui China

**Keywords:** Gestational diabetes mellitus, Glucose, Perinatal depression, Longitudinal study, Repeated data

## Abstract

**Background:**

The association between gestational diabetes mellitus (GDM) and perinatal depression (PND) remains controversial. Our study aimed to comprehensively assess this association in a longitudinal cohort study with repeated measurements of depression.

**Methods:**

Our cohort study was nested in a pilot study of an implementation study aiming to screen and manage perinatal depression within the primary health system in China. Women were recruited in the first trimester from May–September 2019 and followed four times up to 1 year postpartum. Data on sociodemographic characteristics and depression were collected using self-developed questionnaires incorporating the Edinburgh Postnatal Depression Scale (EPDS). Oral glucose tolerance test at 24 ~ 28 weeks and fasting plasma glucose (FPG) data were extracted from medical records. Depression throughout the whole period was divided into different trajectories. Associations of GDM with PND at different time periods and PND of different trajectories were determined by logistic regression. The path of association between blood glucose and depression over time was estimated with an autoregressive cross-lagged model.

**Results:**

In total, 1043 women were included in this analysis and 313 (30.0%) were diagnosed with GDM. The prevalence of depression in the first, second, and third trimesters and postpartum period were 17.2, 6.9, 6.8 and 9.0%, respectively. GDM was neither significantly associated with PND at any time point nor with any specific trajectory of depression. Except for autoregressive paths, no cross-lagged path of FPG and scores of EPDS was significant.

**Conclusions:**

Our study indicates no association between GDM/blood glucose and PND.

**Supplementary Information:**

The online version contains supplementary material available at 10.1186/s12884-022-04667-2.

## Background

Gestational diabetes mellitus (GDM), the most common complication during pregnancy, is defined as glucose intolerance resulting in hyperglycemia that begins or is first diagnosed in pregnancy [1]. Considerable variation in GDM prevalence is observed worldwide, ranging from 6.1 to 15.2% [2]. GDM could pose both short- and long-term harmful effects on mother and their offspring such as preeclampsia, macrosomia, neonatal hypoglycemia and increased lifetime risk of type 2 diabetes by up to 20 times [3–6].

Perinatal depression (PND), a common complication in the perinatal period [7], refers to depression that occurs during a specific period from pregnancy to 1 year postpartum. PND could have both short- and long-term adverse consequences, such as increased risks of abortion, premature delivery, a lower mean birth weight, suicide, infanticide and increased behavioral/emotional problems during childhood [8–10]. Due to differences in screening tools and economic and cultural backgrounds, time periods, the reported prevalence of PND worldwide varies, ranging from 7% ~ 25% [11].

Several possible underlying mechanisms have been suggested in linking diabetes and depression, including inflammation, the hypothalamo-pituitary-adrenal (HPA) axis and psychobehavioral mechanisms [12]. The first paper reporting the relationship between GDM and PND was published in 2009 and indicated prepregnancy or gestational diabetes was independently associated with PND [13]. Continuously, many studies followed but the relationship is still inconclusive, for example, a cross-sectional study from Ethiopia found significant association between antenatal depression and GDM [14], while a cohort study from UK reported that depression during pregnancy was not associated with the risk of GDM [15]. The discrepancy may due to factors such as race, period, depression screening tools, diagnosis criteria of GDM and study design [14–17]. More importantly, PND changes dynamically, with the prevalence varying greatly among different periods [18]. The association between blood glucose and depression should be assessed at multiple time points during the entire perinatal period. Current studies are mostly cross-sectional in nature [14, 19], and the few longitudinal studies on the topic [15, 20, 21] are limited to one or two periods and fail to take a panoramic view of the entire perinatal period.

Thus, nested in an implementation study designed as a longitudinal cohort study collecting repeated measurements during the whole perinatal period, our study aims to comprehensively clarify the associations between GDM and PND. First, we used longitudinal repeated data to determine whether GDM was associated with PND in different periods and whether the trajectories of PND throughout the whole perinatal period differed between the GDM and non-GDM groups. At the same time, we explored the temporal associations between blood glucose and PND using an autoregressive cross-lagged model (ARCLM). Furthermore, as depression and anxiety are strongly correlated [22], we performed a supplementary analysis to assess the associations of GDM between comorbid depression and anxiety.

## Methods

### Design and settings

Our current study was nested in a pilot study of an implementation study, which aimed to create an effective and sustainable perinatal depression screening and management (PDSM) program within the maternal and child health care system in China. If the pilot study in Ma’anshan city succeeded, this program would be scaled-up to three other cities in Anhui province with different socioeconomic levels: Hefei, Bengbu, and Fuyang. Implementation study is the systematic study of how a specific set of activities and designated strategies are used to successfully integrate an evidence-based public health intervention within specific settings [23].

This pilot study of the implementation study adopted a longitudinal cohort design. In total, 1189 women were recruited in the first trimester (< 14^+ 6^ weeks) to collect baseline information. Each woman was followed up in the second (15 ~ 27^+ 6^ weeks) and third (28 ~ 40 weeks) trimesters and within 1 year postpartum. At each time point, depression and anxiety were assessed by the WeChat screening tool. On a voluntary basis, women who were screened as depression positive were provided the internet-based Thinking Healthy Programme (iTHP) intervention. Using data of depression at different time points, and fasting plasma glucose (FPG) and 75-g oral glucose tolerance test (OGTT) extracted from medical records, we conducted a secondary analysis to clarify the relationship between GDM/blood glucose and PND. Due to the impact of COVID-19, the follow-up time during the postpartum period varied, so if multiple evaluations were available, we chose the one closest to 3 months postpartum; otherwise, as long as it was within 1 year postpartum.

### Participants

**Fig. 1:**
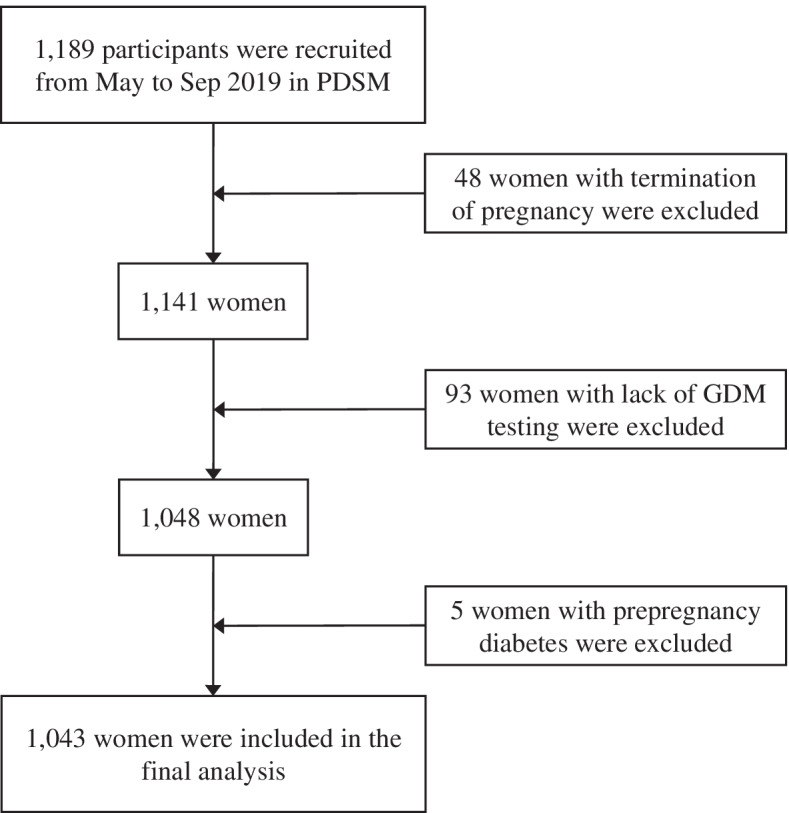
Flow chart of participant inclusion and exclusion of our study

Women in early pregnancy who received prenatal care in Ma’anshan Maternal and Child Health Care Center from May to September 2019 were recruited. The inclusion criteria were as follows: (1) age ≥ 18 years; (2) gestational age ≤ 14^+ 6^ weeks; and (3) completion of questionnaires independently. The exclusion criteria were as follows: (1) termination of pregnancy; (2) diabetes before pregnancy; and (3) lack of GDM testing. The flow chart of the exclusion process is presented in Fig. 1. In total, 1043 women were finally included in this study. The present study obtained ethics approval from the ethics committee of Anhui Medical University (20170358). Written informed consent was obtained from all participating women.

### Measuring tools

#### Sociodemographic information

We compiled a questionnaire (Additional File 1) to collect the sociodemographic information of women. In this study, we applied closed-ended questions and constructed variables including socioeconomic characteristics (e.g., age, annual household income, occupation, education, social status), lifestyle habits (e.g., smoking, passive smoking, drinking), maternal history of pregnancy (e.g., gravidity, parity) and conception (e.g., method, season). Smoking referred to having smoked at least 100 cigarettes. Passive smoking in the past year referred to smoking passively at least once a week. Drinking referred to drinking at least 1 ~ 3 times a month (one drink was defined as up to 340 ml of beer, 140 ml of wine or 43 ml of liquor). The season of conception was designated spring (March, April, May), summer (June, July, August), autumn (September, October, November) or winter (December, January, February). Weight gain per week in the second trimester was calculated using weight and the date of physical examination in the first and second trimesters. Family history of diabetes was defined as the mother or father having diabetes. Data on weight, date of physical examination and parental diabetes were obtained from clinical records.

#### GDM diagnosis

At 24 ~ 28 weeks of pregnancy, 75 g of glucose was orally administered to the pregnant woman for the OGTT in the center. The OGTT was performed in the morning after an overnight fast of at least 8 h. Diagnosis of GDM was made when any of the following plasma glucose values were met or exceeded: fasting: 5.1 mmol/L; 1 h: 10.0 mmol/L; 2 h: 8.5 mmol/L [24]. OGTT results and FPG data in the first, second, and third trimesters and intrapartum period were extracted from electronic patient files.

#### Depression

The Edinburgh Postnatal Depression Scale (EPDS) is a 10-item self-report scale for the evaluation of depression [25]. Participants responded based on their experiences and feelings over the previous week. Each question has 4 scores (0 ~ 3), with possible total scores ranging from 0 ~ 30 and higher scores indicating higher levels of depression. In this population, a cutoff score ≥ 9 was used to categorize women with or without depression [26].

#### Anxiety

The 7-item generalized anxiety disorder scale (GAD-7) is a self-administered questionnaire and was developed in 2006 as a brief screening tool to detect anxiety and assess the severity of anxiety [27]. The score of each item ranges from 0 to 3, and the overall score of the GAD-7 ranges from 0 to 21. Higher scores indicate more severe of anxiety. In this population, a cutoff score ≥ 5 was used to categorize women with or without anxiety [28].

### Statistical analysis

Categorical variables are represented by frequencies and percentages, and continuous data are expressed as the mean ± standard deviation. First, the chi-square test for categorical variables and the t-test for continuous data were used to evaluate differences in characteristics between the non-GDM and GDM groups. Second, binary logistic regression was used to determine the association between GDM and PND by unadjusted or adjusted models. Adjusted covariates were selected based on biologic plausibility, t-tests and chi-square tests, of which *p*-values were less than 0.1. For the first and second trimesters, to assess the association between GDM and PND, binary logistic regression adjusted for covariates related to GDM was used; GDM was treated as the dependent variable and depression as the independent variable. In the third trimester and postpartum period, depression was treated as the dependent variable and GDM as the independent variable after adjusting for covariates related to PND. Third, using the latent class growth model (LCGM), the change in depression was divided into different trajectories according to the EPDS scores throughout the whole perinatal period. Afterward, we used logistic regression to evaluate whether GDM was associated with the trajectory. Finally, an ARCLM was applied to further estimate the path of association between FPG and EPDS over time after adjusting for age and prepregnancy BMI as covariates.

In the supplementary analysis, binary and multinomial logistic regression were used to clarify the association between GDM and comorbid depression and anxiety by adjusting for covariates. Due to some missing data on GAD-7 scores, 1008 women were included in this supplementary analysis. Since some of the women have received iTHP intervention, supplementary analysis of whether iTHP intervention would impact the association between GDM and PND was conducted, by making a comparison of the magnitude of associations of GDM and PND between women who received the iTHP intervention and those who did not. And whether iTHP intervention would impact the incidence of GDM was conducted as well, by making a comparison of GDM incidence between women (depression screened positive) who received the iTHP intervention and those who did not.

Risks are described as unadjusted and adjusted odds ratios (*ORs*) with 95% confidence intervals (*CIs*). All tests were two-sided, and *p*-values less than 0.05 indicated significance. Statistical analysis was carried out using SPSS version 23 and Mplus version 7.4. Version 6 of GraphPad Prism was used to draw forest plots of *ORs* and 95% *CIs*.

## Results

Figure 1 shows the process of including and excluding. After exclusion of 146 women who had termination of pregnancy, lacked a GDM testing or had diabetes before pregnancy, 1043 (87.7%) women were finally included in this study. Follow-up rates in the second and third trimesters and the postpartum period were 88.3% (921/1043), 73.8% (770/1043) and 65.3% (681/1043), respectively. The detection rate of GDM in the second trimester was 30.0% (313/1043). Table 1 shows the characteristics of the participants, comparing those diagnosed with GDM and those without GDM. The age (mean ± SD) of all the participants was 28.76 ± 4.04 years.

**Table 1:** Comparison of baseline characteristics between the GDM versus non-GDM groups

Characteristics	Total sample (***N*** = 1043)	GDM		***p***
		Yes (***n*** = 313)	No (***n*** = 730)	
Age (years)	28.76 ± 4.04	29.60 ± 4.17	28.40 ± 3.92	**< 0.001**
Prepregnancy BMI (kg/m^2^)	21.67 ± 3.36	22.78 ± 3.59	21.19 ± 3.14	**< 0.001**
Weight gain per week in the second trimester (kg/week)	0.44 ± 0.20	0.39 ± 0.21	0.46 ± 0.19	**< 0.001**
Ethnicity				0.446
Han ^a^	1029 (98.7)	307 (98.1)	722 (98.9)	
Other	14 (1.3)	6 (1.9)	8 (1.1)	
Residence				0.250
Urban	915 (87.7)	269 (85.9)	646 (88.5)	
Rural	128 (12.3)	44 (14.1)	84 (11.5)	
Marital status				0.707
Married	982 (94.2)	296 (94.6)	686 (94.0)	
Unmarried or other	61 (5.8)	17 (5.4)	44 (6.0)	
Educational status				0.613
Middle school or below	175 (16.8)	52 (16.6)	123 (16.8)	
High school or technical secondary school	198 (19.0)	64 (20.4)	134 (18.4)	
Junior college or regular college	611 (58.6)	176 (56.2)	435 (59.6)	
Graduate or above	59 (5.6)	21 (6.7)	38 (5.2)	
Annual household income (CNY)				0.906
< 50,000	110 (10.5)	31 (9.9)	79 (10.8)	
50,000 ~ 200,000	788 (75.6)	238 (76.0)	550 (75.3)	
> 200,000	145 (13.9)	44 (14.1)	101 (13.8)	
Occupation				**0.010**
Inoccupation	347 (33.3)	111 (35.5)	236 (32.3)	
Farmers/workers/individuals	129 (12.4)	45 (14.4)	84 (11.5)	
Technical personnel	433 (41.5)	107 (34.2)	326 (44.7)	
Leader/cadre/boss	134 (12.8)	50 (16.0)	84 (11.5)	
Work status				0.501
Resign	427 (40.9)	137 (43.8)	290 (39.7)	
Paid leave	56 (5.4)	13 (4.2)	43 (5.9)	
Part-time job	22 (2.1)	6 (1.9)	16 (2.2)	
Full-time job	538 (51.6)	157 (50.2)	381 (52.2)	
Social status compared with people within the province				0.623
Low (1 ~ 3)	105 (10.1)	31 (9.9)	74 (10.1)	
Medium (4 ~ 6)	695 (66.6)	203 (64.9)	492 (67.4)	
High (7 ~ 10)	243 (23.3)	79 (25.2)	164 (22.5)	
Social status compared with surrounding people				0.626
Low (1 ~ 3)	61 (5.8)	16 (5.1)	45 (6.2)	
Medium (4 ~ 6)	816 (78.2)	243 (77.6)	573 (78.5)	
High (7 ~ 10)	166 (15.9)	54 (17.3)	112 (15.3)	
Conception method				**0.031**
Natural	973 (93.3)	284 (90.7)	689 (94.4)	
Assisted	70 (6.7)	29 (9.3)	41 (5.6)	
Conception season				**< 0.001**
Spring^b^	700 (67.1)	246 (78.6)	454 (62.2)	
Summer	343 (32.9)	67 (21.4)	276 (37.8)	
Unexpected pregnancy				0.911
Yes	221 (21.2)	67 (21.4)	154 (21.1)	
No	822 (78.8)	246 (78.6)	576 (78.9)	
Smoking				0.546
Yes	44 (4.2)	15 (4.8)	29 (4.0)	
No	999 (95.8)	298 (95.2)	701 (96.0)	
Passive smoking in the past year				0.424
Yes	362 (34.7)	103 (32.9)	259 (35.5)	
No	681 (65.3)	210 (67.1)	471 (64.5)	
Drinking				0.986
Yes	143 (13.7)	43 (13.7)	100 (13.7)	
No	900 (86.3)	270 (86.3)	630 (86.3)	
Family history of diabetes				**0.010**
Yes	85 (8.1)	36 (11.5)	49 (6.7)	
No	958 (91.9)	277 (88.5)	681 (93.3)	
Gravidity				**0.008**
1	462 (44.3)	119 (38.0)	343 (47.0)	
≥ 2	581 (55.7)	194 (62.0)	387 (53.0)	
Parity				**0.005**
0	670 (64.2)	181 (57.8)	489 (67.0)	
≥ 1	373 (35.8)	132 (42.2)	241 (33.0)	

Data are presented as n (%) or the mean ± standard deviation

*GDM* gestational diabetes mellitus, *BMI* body mass index

^a^Included 5 missing values

^b^Included 1 woman whose conception date was February 28, 2019

**Fig. 2:**
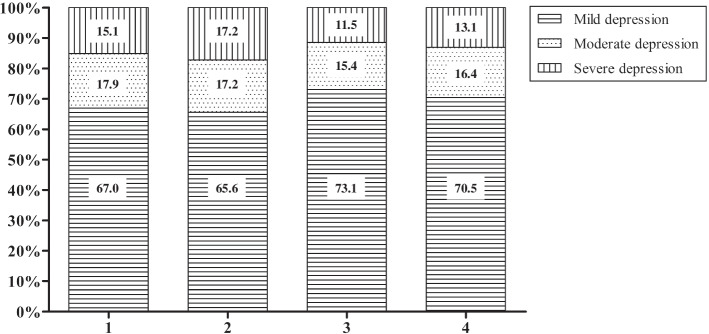
Proportions of different severities of PND. Note: mild depression, EPDS 9 ~ 11; moderate depression, EPDS 12 ~ 13; severe depression, EPDS ≥14; 1, 2, 3 and 4 on the X axis indicate the first, second, and third trimesters and the postpartum period, respectively

The prevalences of depression in the first, second, and third trimesters and the postpartum period were 17.2% (179/1043), 6.9% (64/921), 6.8% (52/770) and 9.0% (61/681), respectively. The composition ratios of depression severity in different periods are shown in Fig. 2. Among women screened as depression positive, more than two-thirds of women had mild depression. Few women had moderate and severe depression.

As shown in Table 2 in the crude model (model 1), compared with that with no depression, the risk of GDM with depression in the first trimester slightly decreased (*OR* = 0.647, *95% CI*: 0.444–0.943, *p* = 0.023). After adjustments for age, prepregnancy BMI, occupation, conception method, conception season, family history of diabetes, gravidity and parity in model 2, the association weakened to marginally significant (a*OR* = 0.656, *95% CI*: 0.443–0.973, *p* = 0.036). After adjustment for weight gain per week in the second trimester was added in model 3, the association shifted to nonsignificant (a*OR* = 0.714, *95% CI*: 0.455–1.120, *p* = 0.143). No significant association between GDM and depression in the second trimester (a*OR* = 1.211, *95% CI*: 0.636–2.306, *p* = 0.560), third trimester (a*OR* = 0.702, *95% CI*: 0.344–1.433, *p* = 0.331) or postpartum period (a*OR* = 0.795, *95% CI*: 0.420–1.507, *p* = 0.483) was observed.

**Table 2:** Relationship between risk of GDM and depression in the first and second trimester

Depression	Total N (%)	GDM		Model 1		Model 2		Model 3	
		Yes n (%)	No n (%)	***OR*** (95%***CI***)	***p***	***OR*** (95%***CI***)	***p***	***OR*** (95%***CI***)	***p***
In the first trimester									
No	864 (82.8)	272 (86.9)	592 (81.1)	Reference		Reference		Reference	
Yes	179 (17.2)	41 (13.1)	138 (18.9)	**0.647 (0.444–0.943)**	**0.023**	**0.656 (0.443–0.973)**	**0.036**	0.714 (0.455–1.120)	0.143
In the second trimester									
No	857 (93.1)	253 (93.7)	604 (92.8)	Reference		Reference		Reference	
Yes	64 (6.9)	17 (6.3)	47 (7.2)	0.864 (0.486–1.533)	0.616	0.975 (0.532–1.787)	0.935	1.211 (0.636–2.306)	0.560

Model 1 is unadjusted

Model 2 is adjusted for age, prepregnancy BMI, occupation, conception method, conception season, family history of diabetes, gravidity and parity

Model 3 is further adjusted for weight gain per week in the second trimester

**Fig. 3:**
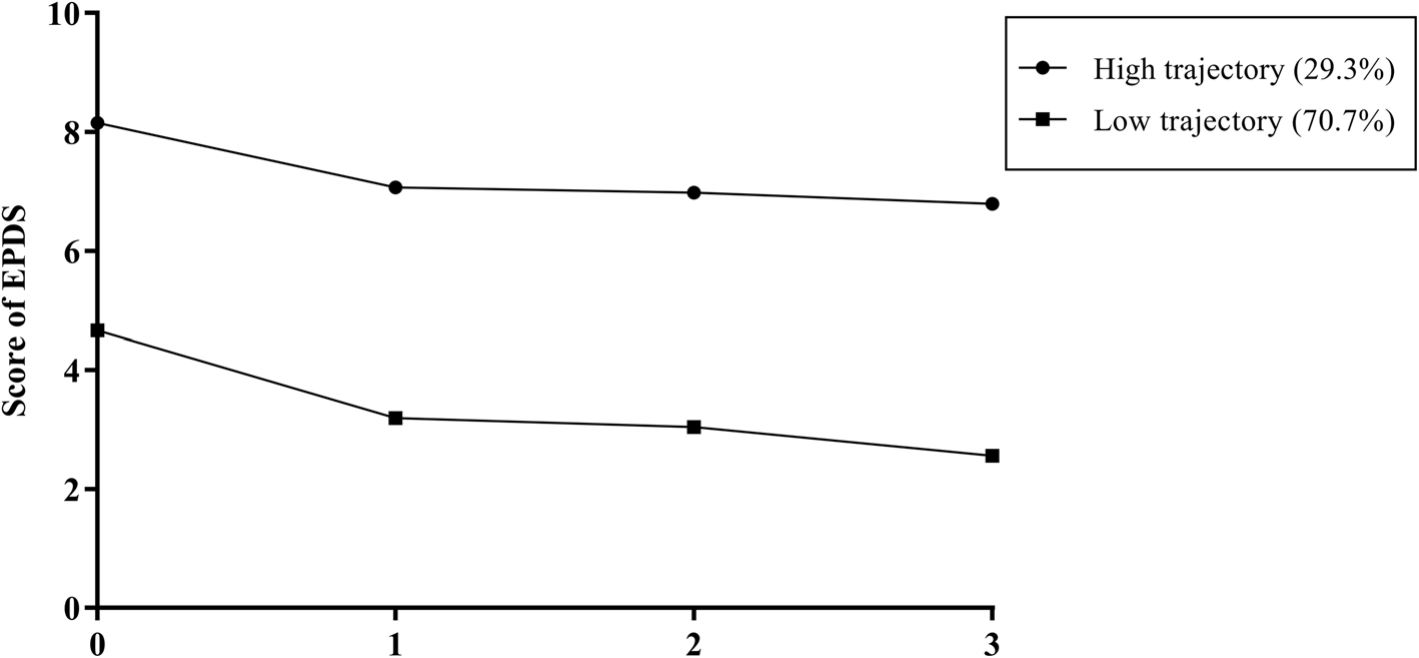
Latent class growth model for 1043 women depending on the EPDS score. Note: 0, 1, 2 and 3 on the X axis indicate the first, second, and third trimesters and the postpartum period, respectively

Based on the EPDS scores of four time points throughout the perinatal period, 1043 women were divided into two trajectory classes by LCGM (as shown in Fig. 3, 29.3% women had a high trajectory, and 70.7% had a low trajectory). The fit index of the model is shown in Table S1. GDM had no influence on the trajectory of PND (a*OR* = 1.092, *95% CI*: 0.797–1.497, *p* = 0.584).

**Fig. 4:**
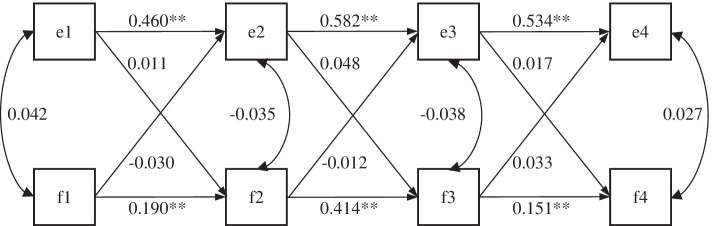
Autoregressive cross-lagged standardized path model of FPG and EPDS. Note: e1, e2, e3 and e4 represent the EPDS scores in the first, second, and third trimesters and postpartum period, respectively; f1, f2, f3 and f4 represent fasting plasma glucose in the first, second, and third trimesters and intrapartum period, respectively; the numbers around the lines represent regression or correlation coefficients; covariates are age and prepregnancy BMI; **p < 0.001, *p < 0.05

Figure 4 shows the standardized model results of ARCLM paths for FPG and EPDS after adjusting for age and prepregnancy BMI. Only autoregressive paths of FPG and EPDS were significant (*p* < 0.001), and no cross-lagged path was significant (*p* > 0.05). No temporal association was observed between EPDS and blood glucose.

The prevalences of the comorbidity of depression and anxiety in the first, second, and third trimesters and the postpartum period were 14.8% (149/1008), 5.4% (47/871), 4.9% (36/730) and 6.9% (45/655), respectively. As shown in Table S2 and Fig. S1, after adjustments were made, no significant association was observed between GDM and depression, anxiety, or their comorbidity at any time point.

Among the 179 women screened as depression positive in the first trimester, 104 accepted the iTHP intervention. As shown in Table S3, no evident differences of characteristics were found between individual receiving intervention and not except for conception season and EPDS scores. No difference of GDM incidence was detected between individuals receiving iTHP and not (a*OR* = 1.535, *95% CI*: 0.609–3.871, *p* = 0.364). No difference (*p* for interaction =0.545) of the association of GDM with PND was observed between individuals receiving iTHP (a*OR* = 0.686, *95% CI*: 0.138–3.419, *p* = 0.646) and not (a*OR* = 1.737, *95% CI*: 0.181–16.673, *p* = 0.632).

## Discussion

Our longitudinal cohort study found no evidence of an association between GDM and PND at any time point throughout the perinatal period, and GDM did not influence the trajectories of PND. Little evidence of a temporal relationship between FPG and EPDS across the perinatal period was found.

In our study, the overall incidence of GDM was 30.0%, which is much higher than the previous report in the same site (12.7%) [29], in mainland China (14.8%) [30] and worldwide (6.1 to 15.2%) [2]. One of the reason for this discrepancy may be the majority of the participants in our current study received OGTT in summer and autumn, which have been proven to be associated with higher chance of GDM diagnosis [31]. The high rate of GDM in our study might impact the extrapolation.

Our study indicated no association between GDM and depression during the whole perinatal period. Our study results are in line with a bunch of studies [15, 32–35]. In addition, FPG in our study was also treated as a continuous variable in the ARCLM, and no association between blood glucose and depression across the perinatal period was revealed which was also supported by a study with large sample size [35]. Unlike us, many studies suggested significant associations between GDM and PND [13, 21, 36–38]. Certain study [13] could not fully distinguish between diabetes and GDM, while in our study, women with prepregnancy diabetes were excluded. In addition, most studies failed to adjust for important potential confounding, such as BMI [13] and seasonality [36], which could bias the association.

After assessing the relationship between GDM and PND in every single period, we explored the association in terms of the whole perinatal period. However, we found that GDM did not influence the trajectory of PND and that FPG had no temporal association with EPDS in the whole perinatal period. To our knowledge, no study has attempted to illustrate the association between GDM and the PND trajectory or the cross-lag effect of PND and FPG. Supplementary analysis results showed no significant association between GDM and the comorbid depression and anxiety at any time point, which is supported by a study from Sichuan, China [39].

To our surprise, a negative association between GDM and depression in the first trimester was found in the crude model. After adjustments for age, prepregnancy BMI, occupation, conception method, conception season, family history of diabetes, gravidity and parity, the association weakened. When further adjustment for weight gain per week in the second trimester was made, the association disappeared. Thus, depression in the first trimester was not independently associated with GDM but might be associated with GDM through one of the adjusted covariates, in particular the weight gain.

A major strength of the present study is the longitudinal cohort design covering the whole perinatal period, and more importantly, rather than only using a binary diagnosis of GDM, our study also including the continuous variable FPG at four time points. Thus, not only could we assess the trajectories of depression but also investigate the temporal association between depression and glucose. Moreover, we further investigated the associations considering comorbid depression and anxiety.

Our study has several limitations. The main limitation of this study is the design of the implementation study with iTHP intervention. The voluntary intervention of iTHP may impact the incidence of GDM and the association between GDM and PND. However, the supplementary analysis indicated neither above mentioned impact. Second, the different characteristics of women who accepted intervention and not may impact the relationship between GDM and PND and the conclusions. Although only conception season and EPDS scores were found to be different in the two groups in the supplementary analysis, there still might be other factors we did not consider, which could influence the results. Besides, previous studies indicated seasonality could influence both GDM and PND [31, 40], thus the relationship between the two might be impacted. Third, in this study, we did not collect preconception EPDS and FPG data which may also impact the risk of both PND and GDM. Fourth, because of COVID-19, the dropout rate during the postpartum period was somewhat high. Additionally, the collection time of the postpartum questionnaires ranged from 42 days to 1 year postpartum; thus, the heterogeneity of the postpartum data should be acknowledged.

## Conclusion

In conclusion, our longitudinal cohort study found no association between GDM or blood glucose and PND.

## Supplementary Information

**Additional file 1.** Socio-demographic Questionnaire. English version of socio-demographic questionnaire.

**Additional file 2. **Supplementary materials for results. Including **Table S1.** (Fit index of latent class growth model), **Table S2.** (Association between risk of GDM, depression and anxiety in the first and second trimesters), **Table S3.** (Comparison of baseline characteristics and EPDS between individual receiving the iTHP and not), and **Figure S1.** (Association between GDM and risk of depression and anxiety in the third trimester and postpartum period).

## Data Availability

Data used in the analysis are available from the corresponding author on reasonable request.
