# Supplementary material for: A longitudinal cohort study of gestational diabetes mellitus and perinatal depression
Source: BMC Pregnancy Childbirth. 2022 Apr 19;22:337. doi: 10.1186/s12884-022-04667-2 (PMC9017412; doi:10.1186/s12884-022-04667-2)
Supplement: Supplementary file 1 — Additional file 1. Socio-demographic Questionnaire. English version of socio-demographic questionnaire. [file 12884_2022_4667_MOESM1_ESM.docx]

**Socio-demographic Questionnaire**

1. Name: ___________________
2. Ethnicity: ___________________
3. ID number: 🞎🞎🞎 🞎🞎🞎 🞎🞎🞎🞎 🞎🞎🞎🞎 🞎🞎🞎🞎
4. Tel: 🞎🞎🞎 🞎🞎🞎🞎 🞎🞎🞎🞎
5. Emergency contact tel: 🞎🞎🞎 🞎🞎🞎🞎 🞎🞎🞎🞎
6. The area you currently live in belongs to:

①Urban; ②Rural

1. Your current marital status:

①Married; ②Divorced; ③Separated; ④Unmarried; ⑤Cohabiting; ⑥Widowed

1. Assuming that the person with the highest socioeconomic status in your province has 10 points (taking income, education and occupation into account together) and the person with the lowest socioeconomic status has 1 point. Lower score means lower status. What score do you think you have? Please tick the appropriate number.


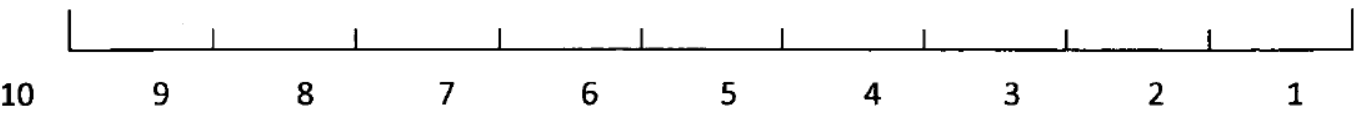


1. Compared with the most people around you (including colleagues, relatives, friends and neighbors), where do you think your socioeconomic status (taking income, education and occupation into account together) is on the following scale? Please tick the appropriate number.


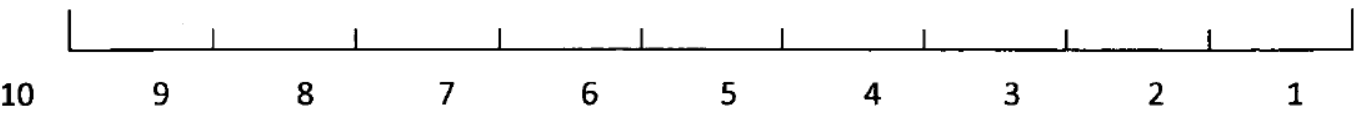


1. Which of the following levels was the total after-tax income of your family last year? (CNY)

①<50,000; ②50,000~99,900; ③100,000~199,900; ④200,000~299,900; ⑤≧300,000

1. How many people’s daily expenses should be paid with the total income of your family? ____________
2. Which of the following is your highest degree?

①Below primary school; ②Primary school; ③Middle school; ④High school or technical secondary school; ⑤Junior college or regular college; ⑥Graduate or above

1. Your occupation:

①No occupation; ②Tricycle driver, porter or babysitter; ③Worker, business service employee, agricultural laborer or small self-employed household that is labor-intensive and no need for technical expertise; ④Rural professional and technical personnel, skilled workers, business service employee with certain skill and expertise or self-employed or professional household; ⑤Professional technician, ordinary clerk or business service employee with high income and quasi-white-collar occupational characteristic; ⑥Middle-level leading cadre or senior management personnel of various enterprises; ⑦Senior professional and technical personnel, ordinary cadre of government department, office staff, leading cadre in rural area, leader of municipal democratic parties or private enterprise owner; ⑧Senior leader or senior intellectual

1. Your current work status:

①Resign; ②Paid leave; ③Part-time job; ④Full-time job

1. Which of the following is your smoking status?

Question description: Smoking means that you have smoked at least 100 cigarettes (about 5 packs of cigarettes) in your life. If not, choose “④Never smoke”.

①Smoke now; ②Used to smoke, but stopped after finding out pregnancy; ③Used to smoke and now have quit (not smoke for at least 3 months consecutively); ④Never smoke

1. Have you experienced passive smoking in your home or work place in the past year?

Question description: Did your family member or work colleague smoke in front of you?

①Never or almost never; ②A few times a year, but less than once a month; ③A few times a month, but less than once a week; ④A few times a week, but less than once a day; ⑤Almost every day

1. Which of the following is your drinking status?

Question description: One drink is defined as up to 340 ml of beer, 140 ml of wine or 43 ml of liquor.

①No drinking; ②Drink occasionally (1~3 times per month); ③Drink frequently (once a week or more)

1. Your pre-pregnancy weight: _______kg; your height: _______cm
2. Date of last menstrual period: 🞎🞎🞎🞎 🞎🞎 🞎🞎
3. In which of the following situations did you conceive this time?

①Completely unexpected pregnancy; ②Go with the flow; ③Well prepared

1. Did you conceive this time naturally or after receiving fertility treatment?

①[Conceive](https://fanyi.so.com/?src=onebox#%20conceive) naturally; ②Conceive naturally during IVF or ICSI treatment; ③Conceive after receiving IVF or ICSI; ④Conceive after receiving other fertility treatments (other than IVF or ICSI)

1. The hospital where you plan to do prenatal examination and delivery in the future (name of the hospital) ____________
2. Are you infertile?

Question description: Infertility refers to not conceiving after one year of regular sex with no contraceptive measures.

①Yes; ②No

1. Do you have the following adverse pregnancy history?

Question description: Such as spontaneous abortion, stillbirth, neonatal death, birth defect.

①Yes; ②No

1. You have conceived time(s) and you have given birth to child (children).
2. Have you ever had a clearly diagnosed mental or psychological disorders? If so, please indicate ___________

Question description: Such as schizophrenia, bipolar and related disorder, depression disorder, anxiety disorder, obsessive compulsive and related disorder, trauma and stress related disorder.

①No, never; ②Yes, it’s cured (no recurrence for a year); ③Yes, it’s not cured yet

1. Have you ever taken psychoactive substances that lead to psychological or physical dependence?

Question description: Including narcotic drugs such as opioids, cocaine and cannabis; psychotropic drugs such as sedative-hypnotics, anti-anxiety drugs and hallucinogens; and other dependence

①No, never; ②Yes, I used to; ③Yes, I still take
