# Supplementary material for: A longitudinal cohort study of gestational diabetes mellitus and perinatal depression
Source: BMC Pregnancy Childbirth. 2022 Apr 19;22:337. doi: 10.1186/s12884-022-04667-2 (PMC9017412; doi:10.1186/s12884-022-04667-2)
Supplement: Supplementary file 2 — Additional file 2. Supplementary materials for results. Including Table S1. (Fit index of latent class growth model), Table S2. (Association between risk of GDM, depression and anxiety in the first and second trimesters), Table S3. (Comparison of baseline characteristics and EPDS between individual receiving the iTHP and not), and Figure S1. (Association between GDM and risk of depression and anxiety in the third trimester and postpartum period). [file 12884_2022_4667_MOESM2_ESM.docx]

**Supplementary materials for results**

**A longitudinal cohort study of gestational diabetes mellitus and perinatal depression**

Haiyan Li, Xiayan Yu, Wenjing Qiang, Mengjuan Lu, Minmin Jiang, Yanyan Hou, Yue Gu, Fangbiao Tao, Beibei Zhu^*^

| **Table S1 Fit index of latent class growth model** | | | | | | |
| --- | --- | --- | --- | --- | --- | --- |
| **Model** | **AIC** | **BIC** | **Adjusted-BIC** | **Entropy** | **Technical 11 (LMR)** | **Technical 14 (BLRT)** |
| Model 1 (1 class) | 17333.934 | 17368.583 | 17346.350 |  |  |  |
| **Model 2 (2 classes)** | **16789.111** | **16843.559** | **16808.621** | **0.694** | **<0.001** | **<0.001** |
| Model 3 (3 classes) | 16629.987 | 16704.235 | 16656.593 | 0.670 | 0.0388 | <0.001 |
| Model 4 (4 classes) | 16529.882 | 16623.929 | 16563.583 | 0.740 | 0.0487 | <0.001 |

| **Table S2 Association between risk of GDM, depression and anxiety in the first and second trimesters** | | | | | | |
| --- | --- | --- | --- | --- | --- | --- |
| **Depression/Anxiety** | **Total**  **N (%)** | **GDM** | | **a*OR* (*95%CI*)** | ***p*** |  |
|  |  | **Yes n (%)** | **No n (%)** |  |  |  |
| In the first trimester |  |  |  |  |  |  |
| No depression nor anxiety | 623 (61.8) | 191 (63.7) | 432 (61.0) | Reference |  |  |
| Only depression | 23 (2.3) | 5 (1.7) | 18 (2.5) | 0.503 (0.136-1.859) | 0.303 |  |
| Only anxiety | 213 (21.1) | 70 (23.3) | 143 (20.2) | 1.325 (0.894-1.964) | 0.160 |  |
| Co-morbidity | 149 (14.8) | 34 (11.3) | 115 (16.2) | 0.806 (0.490-1.328) | 0.398 |  |
| In the second trimester |  |  |  |  |  |  |
| No depression nor anxiety | 719 (82.5) | 210 (82.4) | 509 (82.6) | Reference |  |  |
| Only depression | 10 (1.1) | 3 (1.2) | 7 (1.1) | 1.536 (0.368-6.410) | 0.556 |  |
| Only anxiety | 95 (10.9) | 31 (12.2) | 64 (10.4) | 1.547 (0.913-2.619) | 0.105 |  |
| Co-morbidity | 47 (5.4) | 11 (4.3) | 36 (5.8) | 1.138 (0.519-2.498) | 0.747 |  |
| a*OR* is adjusted for age, pre-pregnancy BMI, occupation, conception method, conception season, family history of diabetes, gravidity, parity and weight gain per week in the second trimester | | | | | | |


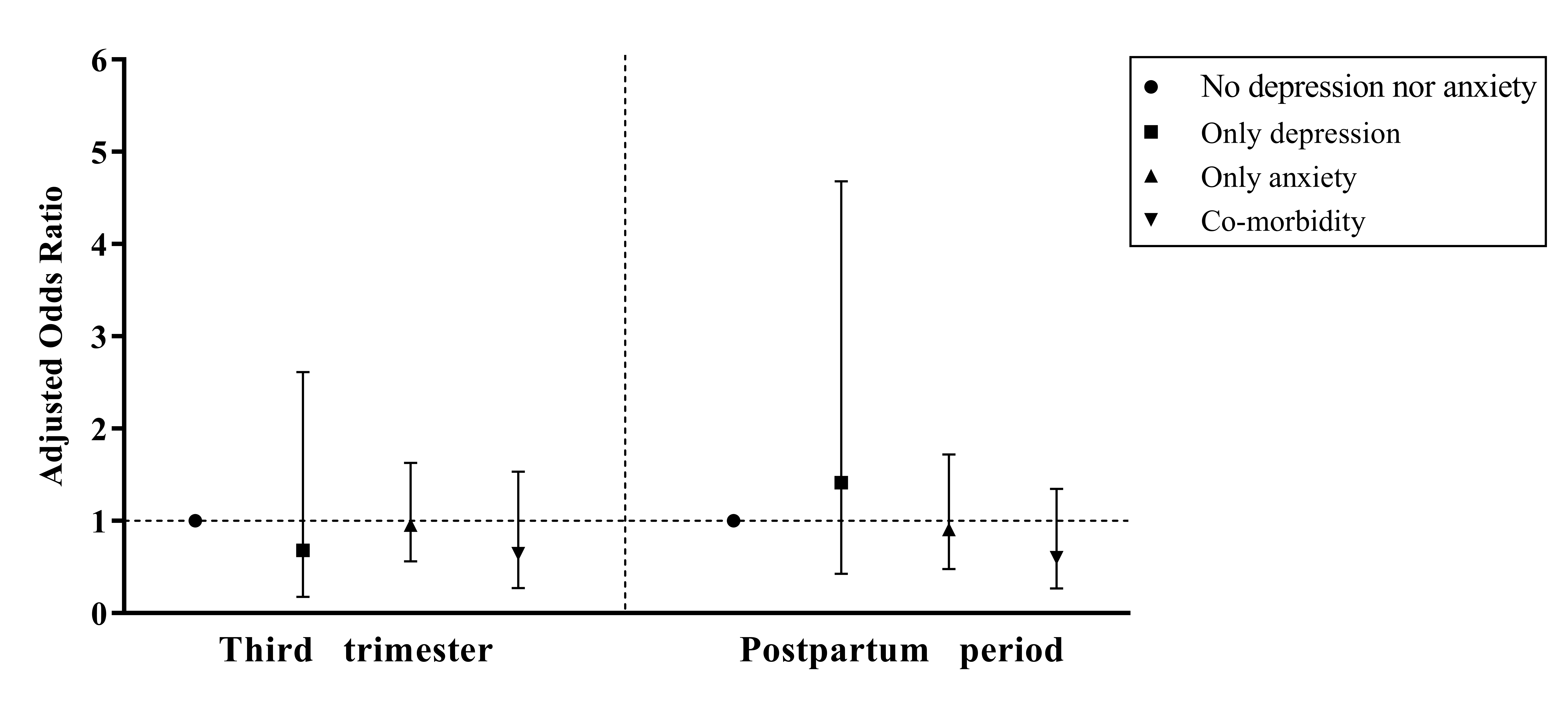


**Fig. S1** Association between GDM and risk of depression and anxiety in the third trimester and postpartum period. Note: a*OR* was estimated from logistic regression models adjusted for age, pre-pregnancy BMI, marital status, work status, social status compared with people surrounding and within the province, unexpected pregnancy and drinking; reference was people with no depression nor anxiety symptoms

| **Table S3 Comparison of baseline characteristics and EPDS score between individual receiving the iTHP and not** | | | |
| --- | --- | --- | --- |
| **Characteristics** | **Yes (n=104)** | **No (n=75)** | ***p*** |
| Age (years) | 28.81 ± 4.16 | 28.38 ± 3.87 | 0.481 |
| Prepregnancy BMI (kg/m^2^) | 21.47 ± 3.11 | 21.57 ± 2.82 | 0.830 |
| Weight gain per week in the second trimester (kg/week) | 0.48 ± 0.20 | 0.42 ± 0.21 | 0.102 |
| EPDS score | 11.59 ± 2.58 | 10.41 ± 1.81 | **<0.001** |
| Ethnicity |  |  | 0.372 |
| Han ^a^ | 101 (97.1) | 75 (100) |  |
| Other | 3 (2.9) | 0 (0) |  |
| Residence |  |  | 0.675 |
| Urban | 91 (87.5) | 64 (85.3) |  |
| Rural | 13 (12.5) | 11 (14.7) |  |
| Marital status |  |  | 0.397 |
| Married | 90 (86.5) | 68 (90.7) |  |
| Unmarried or other | 14 (13.5) | 7 (9.3) |  |
| Educational status |  |  | 0.239 |
| Middle school or below | 19 (18.3) | 21 (28.0) |  |
| High school or technical secondary school | 23 (22.1) | 12 (16.0) |  |
| Junior college or regular college | 55 (52.9) | 40 (53.3) |  |
| Graduate or above | 7 (6.7) | 2 (2.7) |  |
| Annual household income (CNY) |  |  | 0.439 |
| <50,000 | 15 (14.4) | 10 (13.3) |  |
| 50,000~200,000 | 79 (76.0) | 53 (70.7) |  |
| >200,000 | 10 (9.6) | 12 (16.0) |  |
| Occupation |  |  | 0.478 |
| Inoccupation | 44 (42.3) | 34 (45.3) |  |
| Farmers/workers/individuals | 8 (7.7) | 9 (12.0) |  |
| Technical personnel | 41 (39.4) | 22 (29.3) |  |
| Leader/cadre/boss | 11 (10.6) | 10 (13.3) |  |
| Work status |  |  | 0.794 |
| Resign | 53 (51.0) | 39 (52.0) |  |
| Paid leave | 7 (6.7) | 6 (8.0) |  |
| Part-time job | 1 (1.0) | 2 (2.7) |  |
| Full-time job | 43 (41.3) | 28 (37.3) |  |
| Social status compared with people within the province |  |  | 0.245 |
| Low (1~3) | 22 (21.2) | 9 (12.0) |  |
| Medium (4~6) | 67 (64.4) | 56 (74.7) |  |
| High (7~10) | 15 (14.4) | 10 (13.3) |  |
| Social status compared with surrounding people |  |  | 0.699 |
| Low (1~3) | 12 (11.5) | 9 (12.0) |  |
| Medium (4~6) | 78 (75.0) | 59 (78.7) |  |
| High (7~10) | 14 (13.5) | 7 (9.3) |  |
| Conception method |  |  | 0.805 |
| Natural | 98 (94.2) | 70 (93.3) |  |
| Assisted | 6 (5.8) | 5 (6.7) |  |
| Conception season |  |  | **0.004** |
| Spring ^b^ | 59 (56.7) | 58 (77.3) |  |
| Summer | 45 (43.3) | 17 (22.7) |  |
| Unexpected pregnancy |  |  | 0.861 |
| Yes | 72 (69.2) | 51 (68.0) |  |
| No | 32 (30.8) | 24 (32.0) |  |
| Smoking |  |  | 0.574 |
| Yes | 99 (95.2) | 69 (92.0) |  |
| No | 5 (4.8) | 6 (8.0) |  |
| Passive smoking in the past year |  |  | 0.281 |
| Yes | 64 (61.5) | 52 (69.3) |  |
| No | 40 (38.5) | 23 (30.7) |  |
| Drinking |  |  | 0.767 |
| Yes | 81 (77.9) | 57 (76.0) |  |
| No | 23 (22.1) | 18 (24.0) |  |
| Family history of diabetes |  |  | 0.794 |
| Yes | 96 (92.3) | 70 (93.3) |  |
| No | 8 (7.7) | 5 (6.7) |  |
| Gravidity |  |  | 0.202 |
| 1 | 43 (41.3) | 24 (32.0) |  |
| ≥2 | 61 (58.7) | 51 (68.0) |  |
| Parity |  |  |  |
| 0 | 60 (57.7) | 43 (57.3) | 0.962 |
| ≥1 | 44 (42.3) | 32 (42.7) |  |
| Data are presented as n (%) or the mean ± standard deviation  GDM: gestational diabetes mellitus; BMI: body mass index  ^a^ Included 1 missing values | | | |
